# Cofilin-1: A Modulator of Anxiety in Mice

**DOI:** 10.1371/journal.pgen.1002970

**Published:** 2012-10-04

**Authors:** Martin Goodson, Marco B. Rust, Walter Witke, David Bannerman, Richard Mott, Chris P. Ponting, Jonathan Flint

**Affiliations:** 1 Wellcome Trust Centre for Human Genetics, University of Oxford, Oxford, United Kingdom; 2 Neurobiology/Neurophysiology Group, Department of Biology, University of Kaiserslautern, Kaiserslautern, Germany; 3 Institute of Genetics, University of Bonn, Bonn, Germany; 4 Department of Experimental Psychology, University of Oxford, Oxford, United Kingdom; 5 MRC Functional Genomics Unit, Department of Physiology, Anatomy, and Genetics, University of Oxford, Oxford, United Kingdom; University of Chicago, United States of America

## Abstract

The genes involved in conferring susceptibility to anxiety remain obscure. We developed a new method to identify genes at quantitative trait loci (QTLs) in a population of heterogeneous stock mice descended from known progenitor strains. QTLs were partitioned into intervals that can be summarized by a single phylogenetic tree among progenitors and intervals tested for consistency with alleles influencing anxiety at each QTL. By searching for common Gene Ontology functions in candidate genes positioned within those intervals, we identified actin depolymerizing factors (ADFs), including cofilin-1 (Cfl1), as genes involved in regulating anxiety in mice. There was no enrichment for function in the totality of genes under each QTL, indicating the importance of phylogenetic filtering. We confirmed experimentally that forebrain-specific inactivation of Cfl1 decreased anxiety in knockout mice. Our results indicate that similarity of function of mammalian genes can be used to recognize key genetic regulators of anxiety and potentially of other emotional behaviours.

## Author Summary

Thousands of small effect loci are believed to contribute to behavioural variation in mammals. Their abundance and small size frustrate gene identification and make it difficult to know which among them are central to the responsible biological mechanisms. Using imputed genome sequences from 2,000 outbred mice and by testing for an enrichment of functional annotations, we identify 167 candidate genes involved in anxiety. Unexpectedly, annotations implicate actin depolymerizing factors (ADFs), including cofilin-1 (Cfl1), as being involved with the expression of anxiety phenotypes in mice. We confirmed that forebrain-specific inactivation of Cfl1 decreased anxiety in knockout mice.

## Introduction

Exploiting naturally occurring genetic variation to identify mechanisms that give rise to behavioural phenotypes in mammals has proved to be extremely difficult [1]. The abundance and small size of loci that contribute to behavioural variation frustrate gene identification and make it difficult to know which among them are central to the responsible biological mechanisms [2]. A major challenge is to devise methods that move quickly from locus to mechanism [3].

Using heterogeneous stock (HS) mice descended through more than 50 generations from eight inbred progenitor strains [4] we have previously identified 205 quantitative trait loci (QTLs) that contribute to variation in one or more of four anxiety tests: the elevated plus maze, open-field arena, freezing to the context and reluctance to try a novel food. Performance levels on these tests reflect, at least in part, activity in the ventral hippocampus [5], [6], [7], [8], [9] and these tests were chosen in order to interrogate an underlying psychological construct of anxiety from different perspectives (a single measure, such as variation in locomotor activity in the open-field arena, will include traits irrelevant to anxiety [10]). We set out to determine causal genes for anxiety in the HS.

Recombinants that have accumulated since the founding of the HS means that QTLs are mapped to intervals of approximately 3 Mb [4], much higher resolution than obtained by mapping in backcrosses or intercrosses [3]. Nevertheless, mapping in the HS rarely identifies single genes so additional approaches are necessary to identify candidate genes.

Here we considered an alternative approach based on two assumptions. The first is that the mosaic structure of the genomes of inbred laboratory mice could be used to reduce the regions containing candidate genes. Because HS mice are descended from a small number of founders, any pair of mice will share a fraction of their genome [11] so that all genomic regions can be classified as either identical or non-identical by descent. Since a QTL must lie in a region where sequence differences distinguish strains in the same way as the QTL alleles, in a cross between two strains the QTL must lie in a region that is not identical by descent. In multiple crosses, or in animals descended from multiple strains such as the HS, this relationship, though more complex, still holds and can be used to fine-map QTLs [12], [13].

The second assumption is that genes that influence the same, or related, traits have similar functions which are captured by existing functional annotations [14]. The gene ontology (GO) database, for example, assigns biological descriptors (GO terms) to genes [15]. Genes assigned the same GO term can be regarded as members of a category of genes that are more closely related in terms of some aspect(s) of their biology than are randomly-chosen genes. Therefore the presence of a highly non-random pattern of functional annotations is an indication that we have correctly identified genes influencing a trait. Importantly, we do not make any assumptions about which annotations are relevant to a trait prior to performing our analysis.

## Results

### Identification of candidate genes from QTLs: Merge analysis

We used 205 QTLs that contribute to variation in four different anxiety tests: the elevated plus maze, open-field arena, freezing to the context and reluctance to try a novel food. The identification of these QTLs in HS mice is described in [4]. Our first aim was to determine regions within QTLs that are most likely to contain genes involved in the phenotype. To do so, we began by dividing up the genomes of the HS progenitors according to the pattern of ancestral allelic similarities and differences at a locus. Our intention was to identify regions of the genome descended from a common progenitor. Just as each HS mouse is descended from eight inbred strains, the progenitors in turn have ancestors in common. Using a dynamic programming algorithm we partitioned the genome into regions in which all sequence variants detected in the near-complete genome sequence [16] are consistent with a single phylogenetic tree [17].

**Figure 1:**
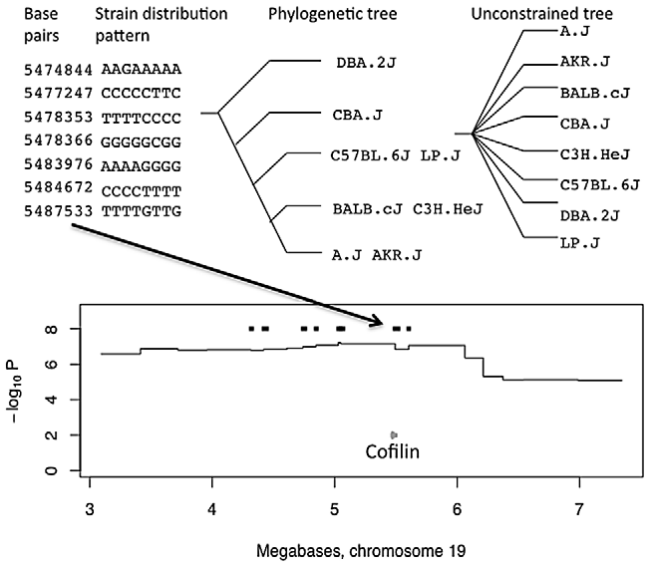
Merge analysis of a QTL on chromosome 19 for open-field activity. The top panel shows the base pair coordinates of seven consecutive single nucleotide polymorphisms. The strain distribution patterns (SDPs) of the variants are shown to the left with strains in the order A/J, AKR/J, BALB/cJ, C3H/HeJ, C57BL/6J, CBA/J, DBA/2J, LP/J, so that for example only BALB/cJ has the G variant at position 5,474,844. The phylogenetic tree derived from these SDPs, is shown next with strain names on the leaves of the tree. The unconstrained tree is shown for comparison. The merge analysis compares the fit of the two trees in explaining the genetic effect at the locus. Regions where the fit of the phylogenetic tree is better than that of the unconstrained tree are retained as consistent QTL intervals, and genes from these regions are used in a GO enrichment analysis. The lower panel shows the position of the consistent QTL intervals (thick black horizontal lines) within the QTL on chromosome 19 influencing open-field activity. The horizontal axis, in megabases, extends over the QTL's 95% confidence interval. The vertical axis gives the negative logarithm (base 10) of the P-value for association between genotype and phenotype. A black arrow connecting top and bottom panels indicates the origin of one consistent QTL, from the relevant strain distribution patterns, that contains the Cofilin gene (grey arrow).

We next used the phylogenetically determined strain distribution patterns to find regions likely to contain genes with variants that could be causally related to phenotypes using a merge analysis [13]. On most phylogenetic trees some founder strains are indistinguishable and so share the same leaf: in other words, the tree merges the eight strains into groups that share alleles. Causal variants lie in intervals where the tree partitions strains consistent with the allelic effects of the QTL. We refer to these intervals as consistent QTL intervals. The 205 anxiety QTLs include 5,932 genes (29 genes per QTL), while the consistent QTLs contain 458 genes (2.4 genes per QTL). Figure 1 presents an example of a merge analysis for a QTL on chromosome 19.

### Functional annotation analysis

Reasoning that causal genes within QTLs for a specific trait are likely to share functions we looked for enrichment of functional annotations (gene ontology (GO) annotations) in the 458 genes within the consistent QTLs. Many tests of functional enrichment assume that the functions of neighboring genes in consistent QTLs are uncorrelated. However neighbouring genes may have similar functions, as tandem duplications, which occur throughout the genome, often give rise to functionally related genes [18]. We addressed this problem using a permutation test that accounts for gene order within genomic intervals. The test assigns P-values to GO annotations, representing the strength of the evidence against the null hypothesis that GO annotations are randomly distributed amongst consistent QTLs.

We summarised the functional coherence of GO annotations associated with the set of genes within consistent QTLs. We define the ‘information score’ as the sum of the negative base-10 logarithms of the P-values (logP) for all GO terms associated with genes within the intervals. The information score can be considered to be a measure of the degree of coherence within the set of genes compared to that in a random sample of genes, and has similarities to the self-information measure of information theory.

For the enrichment analysis we combined genes from all phenotypes, since our aim is to identify biological features that reflect the underlying psychological construct of anxiety, rather than to identify test specific features. The location of QTLs for different measures of anxiety sometimes coincides, for example when we have multiple measures from a single test, such as the open-field arena and elevated plus maze. For overlapping QTLs, we included the QTL with the smallest 95% confidence interval.

**Figure 2:**
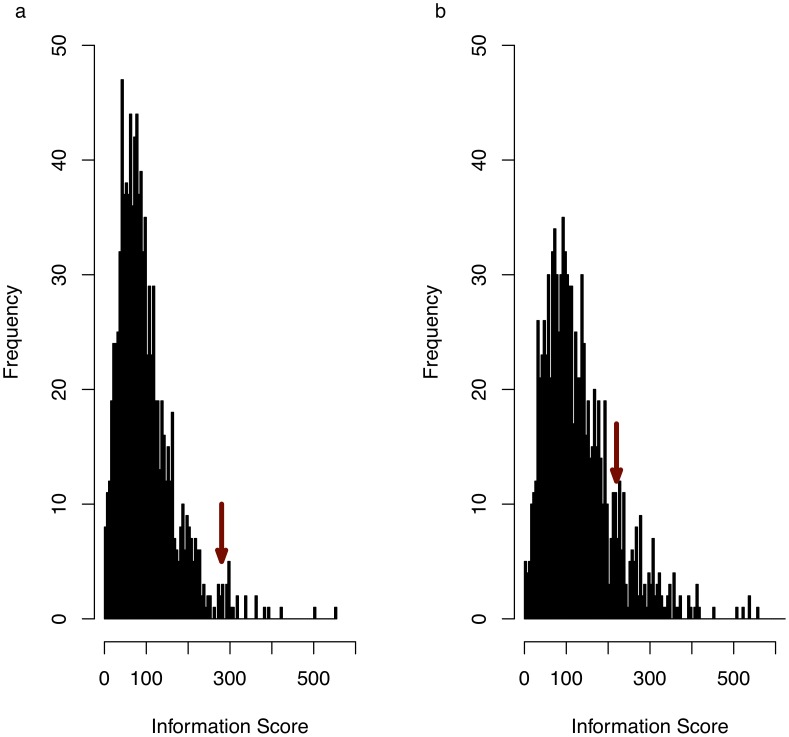
Anxiety associated consistent QTLs define a functionally coherent set of genes. (a) histogram of information scores (see main text for definition) for 5,000 randomly randomly sampled genomic intervals, each with a length distribution identical to that of the anxiety-associated consistent QTLs. Arrow indicates observed information score for experimentally obtained consistent QTLs (P = 0.018). (b) The same analysis as (a) but obtained by including all genes within QTLs, whether or not they overlap a consistent QTL. Arrow indicates information score for experimentally obtained QTLs (empirical P-value = 0.12).

Figure 2A shows a significant enrichment in information score for the genes in the consistent QTL intervals compared to values calculated from 10,000 sets of randomly sampled genes for each of the trait sets. Figure 2B shows that no significant enrichment was found when an identical analysis was performed using all genes at each QTL, ignoring the results of the merge analysis. We tested for over-representation of GO terms, since it is not clear how to validate genes associated with under-represented GO terms (an under-represented gene would be one that is not involved in the phenotype). At a 10% false discovery rate (FDR) we identified 16 GO terms that are over represented in 167 genes at 57 QTLs (Table S1).

More than 90% of the genes were identified by domain-level terms (biological and cellular process) or high-level terms (anatomical structure development, system development, developmental process, multicellular organismal process, cellular metabolic process). Only three GO terms yielded information about specific mechanisms: two genes (cofilin-1 (Cfl1) and destrin (Dstn)) were associated with “positive regulation of actin filament depolymerization’, and a single gene was associated with both “eye pigment granule organization and biogenesis” and “lens morphogenesis in camera-type eye”. However this gene, *Shroom2*, is also associated with the GO term “negative regulation of actin filament depolymerization”, suggesting that actin filament depolymerization might be an important mechanism involved in anxiety.

### Analysis of mutant mice

We examined the effect of disrupting polymerisation/depolymerisation of actin filaments in the hippocampus of mice by using a conditional mutant, n-Cof^flx/flx,CaMKII-cre^, in which Cfl1 is deleted in the principal neurons of the developed forebrain (which includes the hippocampal formation) [19], [20], [21]. Previous work has demonstrated that CamKIIα-cre mice are indistinguishable to wild-type littermates (e.g. [22]–[23]) so we employed littermate mice as controls for the experiments described below.

Anxiety occurs when there is a conflict between competing goals or response options [24], [25]. For example, most unconditioned laboratory tests of anxiety rely on the conflict between whether the animal should approach and explore the relatively more open and exposed sections of the apparatus, or avoid these potentially more dangerous areas. Changes in approach/avoidance behaviour in novel, mildly aversive environments were used as a measure of anxiety, and are dependent, in part, on the ventral hippocampus [5], [6], [7], [8]. An anxious rodent will be slower to enter, and will spend less time in, the more open and exposed sections of the apparatus (e.g. open field arena (OFA) and elevated plus maze (EPM)). They will also defecate when placed in a brightly lit OFA [6]. Numerous studies have used anxiolytic drugs to show a correspondence between the behaviour of rodents in the OFA and EPM and human anxiety [26], [27].

**Figure 3:**
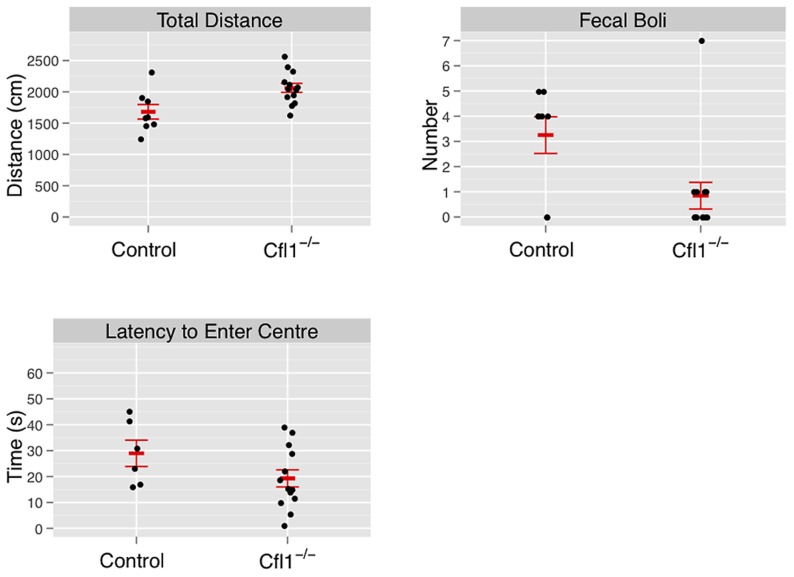
Forebrain-specific deletion of Cfl1 reduces anxiety in the open-field arena. “Clf1^−/−^” indicates n-Cof^flx/flx,CaMKII-cre^ mice; “Control” indicates n-Cof^flx/flx^ controls. Error bars indicate standard error of the mean (sem). (a) Total activity, measured by total distance travelled (controls: 1681 cm (s.e.m. = 116), mutants: 2063 cm, s.e.m. = 72, P = 0.008; adjusted R^2^ = 0.28); (b) defecation (mean number of fecal boli in controls: 3.25 (s.e.m. = 0.73). mutants 0.85 (s.e.m. = 0.53), P = 0.03); (c) latency to enter the center region (control: mean = 42.3 s (s.e.m = 9.5); mutants: mean = 19.3 s (s.e.m. = 3.5); P = 0.01).

**Figure 4:**
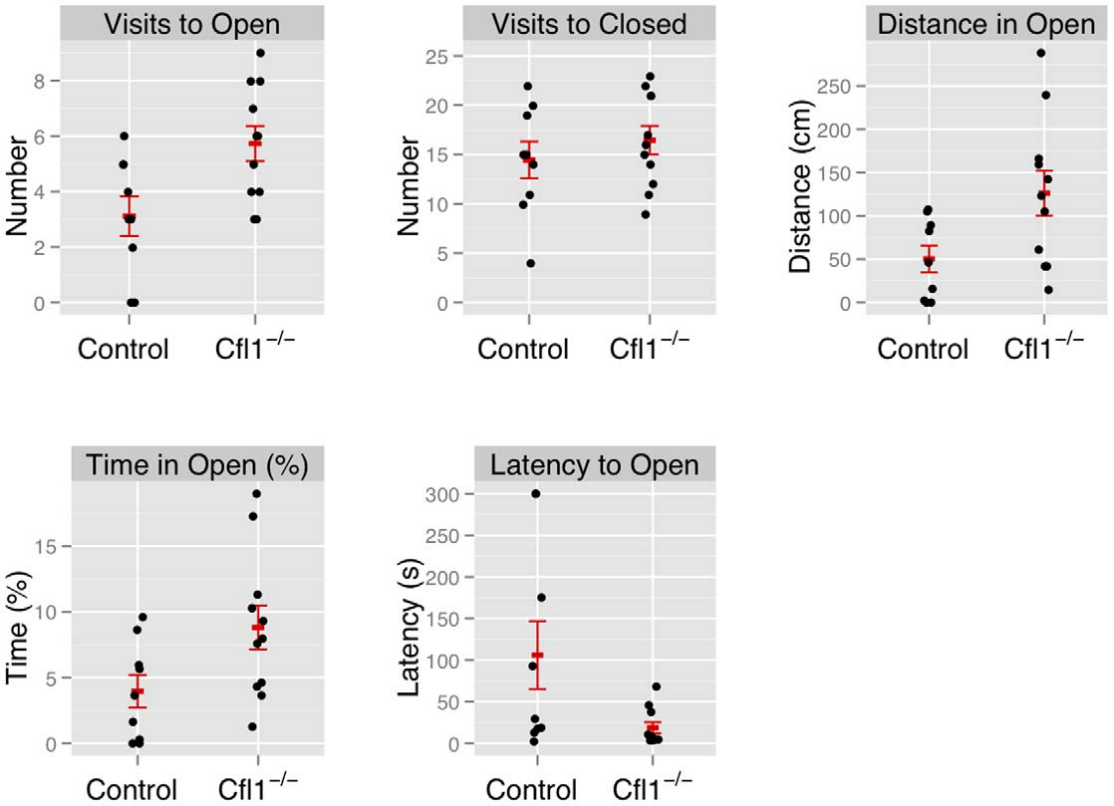
Forebrain-specific deletion of Cfl1 reduces anxiety in the elevated plus maze. n-Cof^flx/flx,CaMKII-cre^ mice in comparison to n-Cof^flx/flx^ controls. Error bars indicate standard error of the mean (s.e.m). (a) Entries into the open arms (mean in controls: 2.88, s.e.m. = 0.77; mean in mutants: 5.73, s.e.m. = 0.63; P = 0.02).). (b) Entries into the closed arms (mean in controls: 13.75 s.e.m. = 1.96; mean in mutants: 16.46, s.e.m. = 1.44; P = 0.32). (c) Distance traveled in the open arms (controls: 46.0 cm, s.e.m. = 17; mutants: 126.2 cm; s.e.m. = 25.8; P = 0.03; adjusted R^2^ = 0.21); (d) Time spent in the open arms, expressed as a percentage of total time (controls: 3.7%, s.e.m. = 1.38; mutants: 8.8%, s.e.m. = 0.63; P = 0.05). (e) Latency to enter the open arms of the apparatus (control: mean = 117.0 s, s.e.m = 44.6; mutants: mean = 18.6 s, s.e.m. = 6.66; P = 0.02).

**Figure 5:**
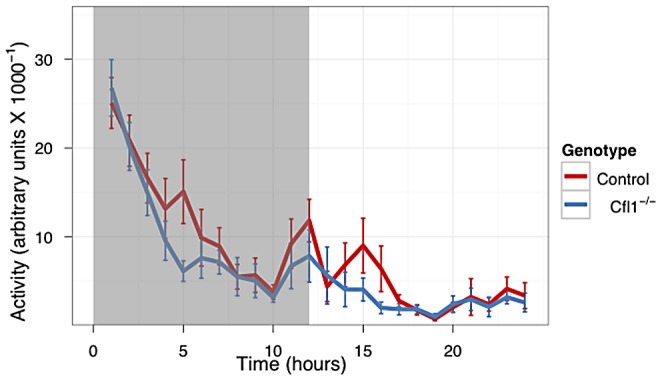
Home Cage activity of n-Cof^flx/flx,CaMKII-cre^ mice was not increased compared to n-Cof^flx/flx^ controls kept on a 12 h dark-light cycle. (n-Cof^flx/flx^ controls n = 18, mean = 8009, n-Cof^flx/flx,CaMKII-cre^ n = 20, mean = 6406. P = 0.33 by Mann-Whitney test).

In both the OFA (Figure 3) and EPM assays (Figure 4) we found that the Cfl1 mutants were significantly less anxious than controls. Mutants showed significantly increased total activity (Figure 3A) and decreased latency to enter the central region in the OFA (Figure 3C). They also defecated less during the OFA test (Figure 3B). The Cfl1 knockouts also spent significantly more time in the open arms of the EPM (Figure 4D). They had longer path lengths within the open arms (Figure 4C), had a reduced latency to enter an open arm for the first time (Figure 4E), and made more entries/visits into the open arms (Figure 4A). Importantly, however, the number of entries into the closed arms of the EPM did not differ between the groups, suggesting that these changes in behaviour do not simply reflect a generalized locomotor hyperactivity in the Cfl1 knockouts (Figure 4B). To explore this further, we examined locomotion of single-housed mutant mice under stress-free conditions using infrared sensors to detect spatial displacement over time in standard mouse cages. In a 24 hour period, neither total activity, nor activity during the light or dark phases, were significantly different between the two genotypes (Figure 5).

## Discussion

In this paper we show how the near-complete sequence from the progenitors of the HS can be use in conjunction with gene annotations to identify genes influencing anxiety at QTLs in HS mice. The method we applied involves partitioning QTLs into intervals that can be summarized by a single phylogenetic tree among the HS founders, testing whether that partitioning was consistent with alleles influencing anxiety at each QTL, and then searching for common functions in candidate genes positioned within those intervals. Crucially, we were able to show there was no enrichment for function when we included all genes under each QTL, thus confirming the value of phylogenetic filtering.

Our method is a development of two analytical techniques, probabilistic ancestral haplotype reconstruction (HAPPY) [28] and merge analysis [13], but it is not a replacement for either; rather, it depends on both. HAPPY is a tool for mapping in populations whose progenitors are known (or can be inferred), while merge analysis identifies which variants might be functional, based on a comparison between the HAPPY derived allelic effects and those of the variant. Incorporating phylogenetic filtering into merge analysis allows us to determine which regions (rather than which variants) are putatively functional and hence to prioritize genes that lie in these intervals for functional studies. Phylogenetic filtering is the methodological advance described here.

Our approach has some obvious limitations. Above all, the relevant gene at most QTLs still remains unknown. At best, we identified genes at 57 QTLs out of 205. Even allowing that the same QTL influences multiple measures, genes at more than half of the QTLs are not identified. This may in part reflect our reliance on an imperfect set of annotations. As the quality and density of annotations increases, it may be possible to detect more functional patterns among genes at consistent QTLs. However failure to find enrichment may also reflect a problem inherent in all sequence based approaches: finding functionally relevant sequence does not immediately translate into finding functionally relevant genes. Typically, as here, genes are identified because they either contain, or lie close to the functionally relevant sequence, but proximity does not unequivocally indentify the correct genes. We were also unable to find many terms that pointed to a potential mechanism. Again this likely reflects the relative poverty of annotations.

Despite these limitations, we think our method has important advantages. Notably it addresses an emerging problem in mouse complex trait, namely the need to prioritize large numbers of candidate genes. Until recently there were relatively few loci mapped at sufficiently high resolution to suggest high quality candidate genes for functional studies. The use of resources that can deliver near gene-level mapping resolution (HS mice, commercial outbreds [29] or the Collaborative Cross [30], [31]), together with the realization that hundreds, if not thousands, of individual genetic variants are involved [32], is about to transform that situation. A critical problem for mouse complex trait analysis problem now is how to validate the large number of candidate genes the new mapping resources identify. The many genes we identified by searching for enrichment of domain-level or high-level GO terms likely provide a useful starting point for functional studies. It should be noted that they include a number of ion channels and neurotransmitter receptors (see Table S1).

Two further observations are worth making about the use of sequence for the identification of Cfl1 as a quantitative trait gene. First, the sequence variants contributing to the QTL likely lie in a regulatory region. From the available progenitor strain sequence we know that no sequence variants segregate in the HS within the Cfl1 gene itself. The nearest 5′ variant is a SNP at 5,489,197 (the transcriptional start site of Cfl1 is at 5,490,455) and the nearest 3′ is a SNP at 5,494,237 (the end of the gene is annotated as 5,494,031). Second, previous mapping of transcript abundance in the HS identified a ci-acting expression QTL for Cfl1 (in the hippocampus) with a logP of 26.4 and a peak at approximately 5.8 Mb ([33] see http://gscan.well.ox.ac.uk/gsBleadingEdge/wwwqtl.cgi). It is possible that the variants contributing to the expression QTL are also those that contribute to the behavioural phenotype (unfortunately we cannot determine whether the alleles in the HS act in the same direction as in the knockout experiment, due to the correlated nature of allelic effects in the HS [4]).

A second issue that warrants discussion relates to the importance of what we have found, namely a relationship between actin filament depolymerisation and genetic differences in anxiety behaviour in the mouse. Since the method depends on gene annotations, we face the objection that we are limited to the discovery of what is already known. Does our work represent an advance in understanding the biology of anxiety?

Rust and co-workers have previously shown that Cfl1 plays an important role in controlling dendritic spine morphology and that the n-Cof^flx/flx,CaMKII-cre^ mice are deficient in long-lasting forms of synaptic plasticity when assessed using hippocampal slices [21]. n-Cof^flx/flx,CaMKII-cre^ mice display behavioural impairments in long-term associative spatial memory [21], as shown by impairment on the standard, spatial reference memory version of the Morris water maze task, in which mice were required to form a long-term association between a particular spatial location and the presence of the escape platform. This raises the possibility that differences in spatial memory abilities and in spatial exploration could have contributed to the observed differences between n-Cof^flx/flx^ and n-Cof^flx/flx,CaMKII-cre^ mice in both the OFA and EPM in the present study. Against this it is important to point out two things.

First, despite their inability to form long-term associative spatial memories, the n-Cof^flx/flx,CaMKII-cre^ mice displayed normal performance on tests of short-term spatial memory [21]. This suggests that the Cfl1 knockout mice are able to discriminate between spatial locations perfectly well, and to acquire this information rapidly. It also shows that these mice do not have a general problem with all aspects of spatial information processing. Second, lesions of the ventral hippocampus have no effect on spatial learning and memory performance. In contrast, lesions of the dorsal hippocampus impair spatial learning and memory but have no effect on tests of anxiety [34]. This double dissociation between the effects of dorsal and ventral hippocampal lesions suggests that the hippocampus may have multiple, dissociable functions associated with different sub-regions of the hippocampus, and that changes in anxiety levels in the Cfl1 knockout mice are unlikely to be due to differences in spatial memory abilities or spatial exploration.

We have assumed here that the effects we observe in the transgenic animals are due to genetic ablation restricted to the hippocampus, but we cannot exclude the involvement of the amygdala. While expression of the Cre recombinase occurs predominantly in the pyramidal neurons of the hippocampus, it also occurs in the striatum, and amygdala [23]. The latter structure is also involved in mediating emotional behaviours, although there appears to be some division of labour between hippocampus and amygdala [35] The anxiety tasks associated with ventral hippocampal lesions are the approach/avoidance tests used here [8], [34], [36] which it is worth noting are generally unaffected by lesions of the amygdala.

Thus we argue that dysfunctional cytoskeletal remodeling and the consequent alterations in synaptic plasticity in the hippocampus, and particularly the ventral hippocampus, are the most likely mechanism that contributes to the altered anxiety levels in Cfl1 knockout mice. Cytoskeletal remodelling is linked to synaptic plasticity and synaptic plasticity is a key neural substrate for emotional behaviours, including anxiety. Indeed, NMDA receptor-mediated synaptic plasticity in the hippocampus is a key determinant of anxiety levels [37]. Anxiety-like states in rodents [38], [39] and humans [40] alter hippocampal dendrites, presumably reflecting synaptic changes. In vertebrates, excitatory synapses are found predominantly on dendritic spines where actin is highly enriched and provides the structural foundation for changes associated with postsynaptic specialization [41]. Electrophysiological measures of synaptic plasticity, long-term potentiation and depression have been associated with growth and shrinkage of dendritic spines respectively [42], [43], [44]. Disruption of genes involved in spine formation can also cause deficits in anxiety behaviour [45] and it has been shown that Lipocalin-2 (Lcn2) regulates stress-induced anxiety in mice via changes in spine morphology and density [46].

Our findings add to this growing literature on the relationship between anxiety-like behaviour and alterations in dendrites. Cfl1 is likely to affect anxiety via the hippocampus, and more specifically the ventral hippocampus. To our knowledge this is the first time that Cfl1 has been implicated as a gene influencing anxiety-like behavior.

## Materials and Methods

### Merge analysis

To take into account the differing degrees of relatedness in the HS we use a mixed models approach where a covariance matrix of the genetic random effects quantifies relatedness in the HS. Variance components were estimated using the R package EMMA [47]. To compare the fit of the strain distribution pattern to the genetic action of the QTL we applied a statistical test, called merge analysis [13]. Merge analysis is related to imputation methods used in human GWAS. It tests whether the strain distribution pattern sequence variants across the HS founders is consistent with the estimated trait values for the founders, by comparing the fit of a QTL linear model in which each founder strain can take a different trait value to one in which those founders sharing the same DNA variant allele are merged and constrained to take the same value. The merge statistic is the negative logarithm of the P-value (logP) of the ANOVA of the merged model. When this value equals, or exceeds the logP of the unmerged model (the unconstrained 8-way haplotype test [28]) the DNA variant could be a QTL allele.

We applied merge analysis with one modification. Our aim was to determine regions likely to contain genes involved in the phenotype, rather than identify the causal variant. So, within the 95% confidence interval for each QTL, we segmented the locus into intervals between the SNPs in the HS mice, based on the ancestral recombination graph among the eight HS progenitors. Within each interval all sequence variants detected in the Sanger mouse genomes database [16] are consistent with the same ancestral tree (i.e. every pair of variants obeys the 4-gamete test). This method is described in [17]. On most trees some founder strains are indistinguishable so share the same leaf. Therefore we used the tree to represent each interval in the merge analysis, by generating a pseudo multi-allelic marker whose alleles correspond to the leaves, and comparing the fit of the tree to the 8-way haplotype test. We designate a merge interval as one whose logP value equals or exceeds the logP of the haplotype test. Thus the merge intervals act as an importance filter on the QTL intervals, subdividing each QTL into regions that could contain causal variants, and therefore are more likely to contain the causal genes. The test was coded in R as an extension to the R HAPPY package (http://www.well.ox.ac.uk/rmott/happy).

### Identification of functional genes using Gene Ontology annotations

If the merge intervals were more likely to contain causal genes than the QTL intervals as a whole, we would expect them to be enriched for certain classes of genes. We tested for over-representation of gene function annotations within the merge intervals. Our null hypothesis is that the merge analysis places intervals randomly within QTL intervals, rather than correctly identifying causal variants for the trait being investigated. However we have to take into account a number of potential biases. First, there may be a bias due to chromosomal location or G+C content of the QTLs. Our sampling procedure therefore draws sampled intervals matched both for chromosome and G+C content. Second, enrichment in GO descriptors could simply be due to larger numbers of genes found within the intervals. We used a procedure that matched the gene numbers within the random intervals to that of the QTL intervals to avoid this type of bias. Finally, tests that assume independence, such as the hypergeometric test, may not provide robust estimates because neighbouring genes within a QTL may have similar functions (for example, functionally similar genes arising from tandem duplications).

We employed a Monte Carlo simulation method to test for over-representation of gene function annotations within genomic intervals. This method does not assume that the function of each gene sampled is independent. The Monte Carlo simulation first identifies all genes that overlap QTLs. For each biological process GO annotation term *j*, we counted the number of QTLs (*n_Qj_*) that overlap any of the genes associated with that annotation.

To identify GO terms that are significantly enriched among genes within consistent QTLs, we created a null distribution from 5,000 sets of randomly sampled genomic intervals, each with a length distribution identical to that of the test set of QTLs. Each set was drawn from regions of similar nucleotide (G+C) content. Chromosomes were divided into 1 Mb-sized windows and each window assigned to one of 10 equally populated %G+C bins. For each of the test QTLs we picked a random genomic location from the same chromosome that was located in a genomic window from the corresponding %G+C bin. Each randomly sampled interval was overlaid with a set of simulated consistent QTL intervals identical to that of the test QTL. For each GO term *j*, the number of randomly sampled regions *n_rj_* that overlap genes associated with *j* was calculated, ignoring genes outside the simulated consistent QTLs. The fraction of these 5,000 sets for which *n_rj_*≥*n_Qj_* is *p_j_*, which represents an estimate of the probability that annotation *j* is observed in *n_Qj_* QTLs simply by chance.

A further 5,000 sets of randomly sampled regions (defined as above) were used to determine the experimental false discovery rate (FDR). For each set, the number of significantly over-represented annotation terms was recorded. The P-value threshold giving the desired FDR value was then applied to the results for downstream analysis.

### Analysis of mutant mice

Gene targeting of the Cfl1 gene was performed in 129 Sv embryonic stem cells [19], [20]. The conditional Cfl1 allelle was backcrossed onto a C5BL6/J background for more than 20 generations. Inactivation of Cfl1 in the principle neurons of the adult forebrain was achieved by crossing a CamKIIα-cre transgene onto the conditional Cfl1 strain [20], [21], [23]. Behavioral analyses were performed on male mice using age-matched littermates (*n-cof^flx/flx^*) as controls. A first cohort of 6–7 week old mice was tested first in the open-field and next in the elevated plus maze. A second cohort of 8–10 week old mice was used for activity recording in a home cage. Mice were housed in an animal facility with 12-hour light-dark cycle and water and food access *ad libitum*. Animal treatment and care were provided in accordance with institutional guidelines.

Home cage locomotion of single-housed mice was assessed in standard mouse cages (Type II) using TSE InfraMot infrared sensors (TSE Systems, Bad Homburg, Germany). Mice were transferred to new cages 12 hours before starting the recordings.

Anxiety was assessed in mice using two different, ethological, unconditioned tests of anxiety. These were the open field arena (OFA) and the elevated plus maze (EPM). A standard rectangular OFA with 0.5 m side length (TSE Systems, Bad Homburg, Germany) was used. At the beginning of the experiment, mice were placed in one of the corners facing away from the center region. Locomotor activity and latency of entering the center region were assessed using the VideoMot2 video tracking system (TSE Systems, Bad Homburg, Germany). Fecal boli were counted manually at the end of each experiment. A T test was used to compare locomotor activity; the Mann-Whitney test was used to compare fecal boli counts and the Mantel-Haenszel test implemented in the ‘survival’ package was used to compare latency of entering the central area. n = 8 for Cfl1 mutants; n = 13 for controls.

A pale grey polyvinyl chloride EPM with the following dimensions was used: arm (either open or closed) length: 300 mm, width 50 mm, and height of the closed arms 150 mm. At the beginning of the experiment, mice were placed in a closed arm facing away from the center region. Time spent in open arms, visits to open and closed arms and distance travelled in open arms were assessed using the VideoMot2 video tracking system (TSE Systems, Bad Homburg, Germany). Latency to enter the open arm was recorded manually. Statistical tests were performed using the R statistical package; the Mann-Whitney test was used to compare the percentage time spent in open arms and the number of visits to open arms. n = 8 for Cfl1 mutants; n = 11 for controls.

### Ethics statement

All animal work was conducted according to UK guidelines and approved by the UK Home Office.

## Supporting Information

Table S1 The table provides information on each of the QTLs for which we observed enriched GO term annotations. The phenotype and location of the QTL are given in the first three columns, followed by the GO term ID, description and fold enrichment. The subsequent columns give the genes associated with each GO term.
(XLSX)
